# Comparative Value of Cod-Liver Oil and Fish Oil Mixed with Iodine

**Published:** 1852-02

**Authors:** 


### MATERIA MEDICA AND THERAPEUTICS.

*Comparative Value of Cod-liver Oil and Fish Oil mixed with Iodine.*—Dr. Champouillon, professor at the Army Medical School of Val de Grace, has just laid before the Academy of Medicine the result of the comparative experiments he has made upon phthisical patients with cod-liver oil, and simple fish oil mixed with iodine. Dr. Champouillon gave the cod-liver oil to 120 patients laboring under phthisis. Fifty-one were in the first stage; and of these, twenty-four were benefitted, and none died. Thirty-seven were in the second stage; of these, nine recovered, and three died. Fourteen were in the third stage; and here six recoveries and four deaths took place. The author gave the iodated oil to seventy-five patients in different stages of phthisis: no improvement took place in any case, and in several it was noticed that the remedy did harm.—*Lancet.*

